# State-dependent central synaptic regulation by GLP-1 is essential for energy homeostasis

**DOI:** 10.21203/rs.3.rs-3929981/v1

**Published:** 2024-03-12

**Authors:** Le Wang, Rohan Savani, Matteo Bernabucci, Yi Lu, Ishnoor Singh, Wei Xu, Abdelfattah El Ouaamari, Michael B. Wheeler, Harvey J. Grill, Mark A. Rossi, Zhiping P. Pang

**Affiliations:** 1 The Child Health Institute of New Jersey, Rutgers Robert Wood Johnson Medical School, New Brunswick, NJ, USA.; 2 Department of Physiology, University of Toronto, Ontario, Canada; 3 Department of Neuroscience, UT Southwestern Medical Center, Dallas, TX, USA; 4 Department of Cell Biology and Anatomy, New York Medical College, Valhalla, NY, USA; 5 Department of Psychology, University of Pennsylvania, Philadelphia, PA, USA; 6 Department of Psychiatry, Rutgers Robert Wood Johnson Medical School, New Brunswick, NJ, USA.; 7 Brain Health Institute, Rutgers University, New Brunswick, NJ, USA; 8 Department of Neuroscience and Cell Biology, Rutgers Robert Wood Johnson Medical School, New Brunswick, NJ, USA.; 9 Department of Pediatrics, Rutgers Robert Wood Johnson Medical School, New Brunswick, NJ, USA.

## Abstract

Central nervous system (CNS) control of metabolism plays a pivotal role in maintaining energy homeostasis. Glucagon-like peptide-1 (GLP-1, encoded by *Gcg*), secreted by a distinct population of neurons located within the nucleus tractus solitarius (NTS), suppresses feeding through projections to multiple brain targets^1–3^. Although GLP-1 analogs are proven clinically effective in treating type 2 diabetes and obesity^4^, the mechanisms of GLP-1 action within the brain remain unclear. Here, we investigate the involvement of GLP-1 receptor (GLP-1R) mediated signaling in a descending circuit formed by GLP-1R neurons in the paraventricular hypothalamic nucleus (PVN^GLP-1R^) that project to dorsal vagal complex (DVC) neurons of the brain stem in mice. PVN^GLP- 1R^→DVC synapses release glutamate that is augmented by GLP-1 via a presynaptic mechanism. Chemogenetic activation of PVN^GLP-1R^→DVC neurons suppresses feeding. The PVN^GLP-1R^→DVC synaptic transmission is dynamically regulated by energy states. In a state of energy deficit, synaptic strength is weaker but is more profoundly augmented by GLP-1R signaling compared to an energy-replete state. In an obese state, the dynamic synaptic strength changes in the PVN^GLP-1R^→DVC descending circuit are disrupted. Blocking PVN^GLP-1R^→DVC synaptic release or ablation of GLP-1R in the presynaptic compartment increases food intake and causes obesity, elevated blood glucose, and impaired insulin sensitivity. These findings suggest that the state-dependent synaptic plasticity in this PVN^GLP-1R^→DVC descending circuit mediated by GLP-1R signaling is an essential regulator of energy homeostasis.

GLP-1 is secreted by L-cells in the small intestine and a discrete population of neurons located in the NTS^1^ and plays a significant role in controlling food intake and maintaining glucose homeostasis^4^. GLP-1 analogs are clinically effective in treating type 2 diabetes and obesity^4^, but they have been reported to be associated with adverse events, including nausea, vomiting, diarrhea, and gallbladder issues^4–6^. The lack of a complete mechanistic understanding of GLP-1R signaling in both the peripheral nervous system and the CNS impedes the development of more effective clinical interventions. The peripheral potentiation of insulin release does not explain the weight loss associated with GLP-1 analogs in humans^4^. Because central and peripheral GLP-1R signaling suppress feeding via independent gut-brain pathways^7^, it is appealing to develop specific strategies to activate only the central GLP-1R signaling pathway to treat obesity via suppression of feeding^1,4^. In the brain, it has been demonstrated that GLP-1R signaling can enhance excitatory presynaptic release in the lateral hypothalamic area^8^ and the paraventricular hypothalamic nucleus (PVN)^9^, two brain regions that are essential in regulating food intake. Ablation of GLP-1R in the PVN leads to obesity and hyperphagia^9^. It has also been shown that NTS GLP-1R signaling plays a role in regulating food intake^10^. However, the role of GLP-1R signaling in PVN→brainstem descending pathways remains uninvestigated. Dissecting the CNS synaptic and neurocircuitry basis of endogenous central GLP-1 release and subsequent function in the regulation of metabolism will contribute to understanding the regulation by GLP-1 in the brain and will also provide an opportunity to further improve GLP-1 analog therapeutics^4^.

## GLP-1R signaling enhances PVN^GLP-1R^→DVC presynaptic release

To identify the downstream neural targets of PVN^GLP-1R^ expressing neurons, we conducted anterograde trans-synaptic tracing. We injected the newly developed anterograde trans-synaptic tracer AAV-Y444F-CAG-DIO-mWGA-mCherry^11^ in GLP-1R-ires-Cre mice^12^ (**Extended Data Fig. 1a&b**) and conducted whole-brain mapping. Among many brain structures, we observed dense expression of mCherry (axon terminals from PVN^GLP-1R^ neurons and postsynaptic expression^11^) in DVC, including the area postrema (AP), NTS, and dorsal motor nucleus of the vagus (DMV). Postsynaptic mCherry+ neurons in the DVC were found to express choline acetyltransferase (ChAT+) in the DMV and tyrosine hydroxylase (TH^+^) in the NTS (**Extended Data Fig. 1c&e**). Additionally, we detected PVN^GLP-1R^ anterograde signals in the median preoptic nucleus (MnPO) and median eminence (ME), consistent with previous studies^13^ (**Extended Data Fig. 1d**). Among all downstream neural targets, the DVC appears to receive the most prominent inputs from PVN^GLP-1R^ neurons (**Extended Data Fig. 1c**). The DVC is a key central region in regulating metabolism^14,15^ and mediating GLP-1R signaling induced food intake suppression^16^. To further verify this PVN^GLP-1R^→DVC descending projection, we performed complementary retrograde tracing by injecting AAVrg-hSyn-DIO-EGFP in the DVC of GLP-1R-ires-Cre mice and systematically quantifying retrogradely labeled PVN^GLP-1R^→DVC neurons (**Extended Data Fig. 1f&g**). PVN^GLP-1R^→DVC neurons are mainly located in the posterior PVN (**Extended Data Fig. 1h**).

**Figure 1:**
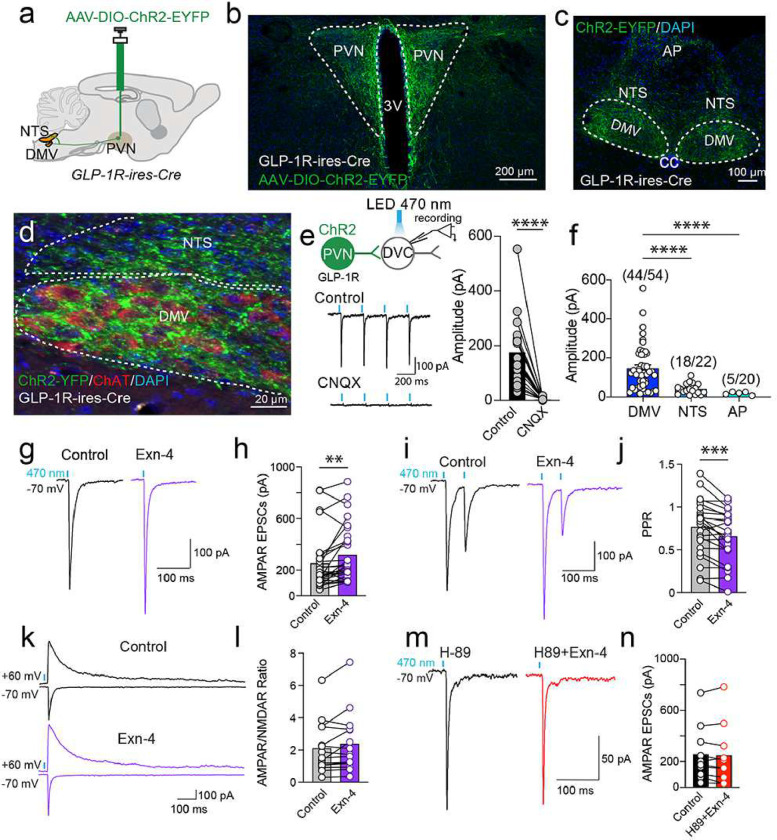
PVNGLP-1R→DVC descending circuit is regulated by GLP-1R-mediated signaling. **a**. Experimental paradigm for AAV-DIO-ChR2-EYFP injection to label PVN GLP-1R neurons. **b**. Representative image of the PVN with AAV-mediated expression of ChR2-EYFP. **c-d**. Representative image of DVC with expression of ChR2-EYFP. **e**. DVC cells were patched and evaluated for synaptic connectivity with optogenetically evoked EPSCs (oEPSCs) that are blocked by CNQX (n=20 cells/12 mice). **f**. Percentage of neurons showing synaptic connections (numbers indicate the numbers of responsive cells/total cells). **g-h**. Representative traces and quantification of AMPAR mediated oEPSCs before and after application of Exn-4 (n=29 cells/12 mice). **i-j**. Representative traces and quantification of light-evoked PPR before and after application of Exn-4 (n=24 cells/12 mice). **k**. Representative traces of oEPSCs with or without Exn-4. AMPAR- and NMDAR-mediated oEPSCs were recorded at holding potentials of −70 mV and +60 mV, respectively. NMDAR- oEPSCs were measured at 50 ms after stimulations. **l**. Pooled data of AMPAR/NMDAR-EPSCs ratio before and after application of Exn-4 (n=15 cells/12 mice). **m-n**. Representative traces and quantification of AMPAR-mediated oEPSCs before and after application of H89 and Exn-4 (n=10 cells/3 mice). Data are presented as mean ± SEM and sample sizes are indicated in each plot; paired student’s t-tests are applied to (**e**) and (**g-n**). One-way ANOVA is applied to (**f**); ∗p< 0.05; ∗∗p< 0.01; ∗∗∗p< 0.001; ∗∗∗∗p< 0.0001.

We then asked if PVN^GLP-1R^ neurons form synapses with DVC neurons using Channelrhodopsin-2 (ChR2) assisted circuit mapping. We injected AAV-DIO-ChR2-EYFP into the PVN of GLP-1R-ires-Cre mice and conducted synaptic physiology in the DVC neurons. Consistent with results from tracing experiments (Fig. 1a–c), we observed dense axon terminals in the DVC, especially the DMV as visualized with ChAT immunostaining (Fig. 1d). Using whole-cell patch clamp electrophysiology, we recorded robust optically evoked excitatory postsynaptic currents (oEPSCs) that were blocked by the AMPA receptor blocker cyanquixaline (CNQX) (Fig. 1e), indicating that PVN^GLP-1R^ neurons form glutamatergic synapses onto DVC neurons. Comparing oEPSC amplitude and the connectivity ratio within the DVC, our data indicate that the DMV receives stronger PVN^GLP-1R^ synaptic input compared to the NTS and AP (Fig. 1f).

Since PVN^GLP-1R^ neurons project to the DVC and release glutamate, we hypothesized that GLP-1 signaling regulates synaptic release at this synapse via presynaptically expressed GLP-1Rs. To test this hypothesis, we recorded PVN^GLP-1R^→DVC oEPSCs in the DMV neurons with or without the GLP-1R agonist Exendin-4 (Exn-4)^17^(**Extended Data Fig. 2a**). PVN^GLP-1R^→DVC oEPSCs are significantly augmented by Exn-4 (Fig. 1g&h). Consistent with our hypothesis of a presynaptic mechanism, paired-pulse ratios (PPR) of oEPSCs in the presence of Exn-4 were reduced (Fig. 1i&J), suggesting an increased presynaptic release probability. To further support this, we also recorded AMPAR and NMDAR oEPSCs, at holding potentials of −70 mV and +60 mV, respectively, and calculated the ratio of AMPAR/NMDAR oEPSCs. No differences were found before and after the application of Exn-4 (Fig. 1k&l), once again indicating presynaptic regulation of synaptic release probability. Moreover, we also observed an increase in spontaneous synaptic release frequency but not amplitudes (**Extended Data Fig. 2b&c**). Collectively, these data provided strong evidence that GLP-1R signaling presynaptically augments PVN^GLP-1R^→DVC synaptic release.

Furthermore, since we previously showed that GLP-1R activates protein kinase A (PKA) to regulate synaptic transmission in the PVN^18^, we hypothesized that a similar GLP-1R signaling cascade is involved in regulating presynaptic release probability. Indeed, we found blocking PKA signaling with H-89 abolished the augmentation of PVN^GLP-1R^→DVC synaptic release by Exn-4 (Fig. 1m&n). These data demonstrate robust synaptic connectivity between PVN^GLP-1R^ neurons and brain stem DVC neurons and suggest that this descending neural pathway may mediate GLP-1R signaling in the brain.

## Activation of PVN^GLP-1R^→DVC suppresses feeding

**Figure 2:**
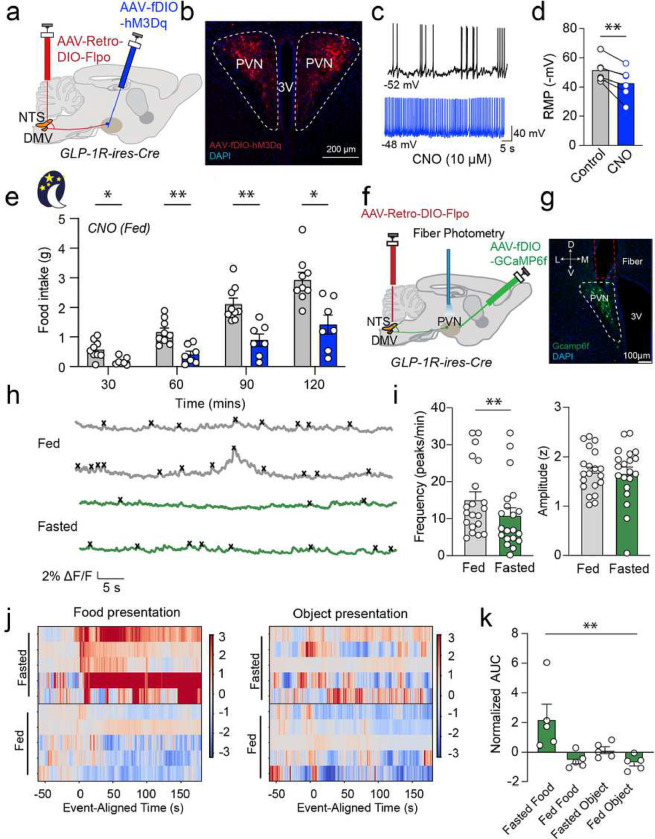
State-dependent PVNGLP-1R→DVC neuronal activity suppresses feeding. **a**. Experimental paradigm for virus delivery and chemogenetic stimulation of PVN^GLP-1R^**→**DVC neurons. **b**. Representative image of AAV-fDIO-hM_3_Dq-mCherry expression in the PVN^GLP-1R^→DVC neurons. **c**. Representative traces of CNO application. **d**. Quantification of CNO application in PVN hM_3_Dq- or control-virus expressing cells (n=6 cells/2 mice). **e**. Food intake consumption upon activation of PVN^GLP-1R^**→**DVC neurons in the dark cycle (control n=9 mice, hM_3_Dq n=7 mice). **f**. Experimental paradigm for virus delivery and fiber photometry imaging of PVN^GLP-1R^**→**DVC neurons. **G**. Representative image of Gcamp6f expression in the PVN^GLP-1R^**→**DVC neurons. **h**. Representative traces of PVN^GLP-1R^**→**DVC neuron’s calcium activity in the fasted and fed state. **i**. Frequency and amplitude quantification of PVN^GLP-1R^**→**DVC neurons’ calcium activity in the fast and fed state (n=20 trial/5 mice). **j-k**. Calcium signals of PVN^GLP-1R^**→**DVC neurons during different food/object presentations and under different energy states (n=5 mice). Data are presented as mean ± SEM and sample sizes are indicated in each plot; paired student’s t-test is applied to (**d**) and (**i**); One-way ANOVA is applied to (**k**). Two-way ANOVA with Geisser-Greenhouse correction is applied to (**e**). *p< 0.05; **p<0.01.

We previously showed that activation of GLP-1 input in the PVN suppresses food^18^. It is unclear whether the activation of PVN^GLP-1R^→DVC neurons is sufficient to inhibit food intake. To this end, we used Designer Receptors Exclusively Activated by Designer Drugs (DREADDs) chemogenetics^19^. To allow robust expression of hM_3_Dq DREADDs in PVN^GLP-1R^→DVC neurons, we injected AAVrg-EF1a-DIO-Flpo in the DVC and AAV-hSyn-fDIO-hM3D(Gq)-mCherry in the PVN of GLP-1R-ires-Cre mice (Fig. 2a&b). The application of clozapine-N-oxide (CNO, 10 μM) resulted in depolarization of membrane potential and increased action potential firing rates in hM_3_Dq-expressing PVN^GLP-1R^ neurons in brain slices (Fig. 2c&d). Chemogenetic activation of PVN^GLP-1R^→DVC neurons in behaving animals with 1mg/kg CNO profoundly suppressed food intake regardless of light- or dark-cycle and energy state (Fig. 2e and **Extended Data Fig. 3b&c**). Mice injected with the vehicle control (saline) showed no significant difference in food intake (**Extended Data Fig. 3a**). The suppression of food intake after chemogenetic activation of PVN^GLP-1R^→DVC neurons did not result from increased anxiety or impaired locomotion (**Extended Data Fig. 3e-i**). Moreover, we also investigated whether activation of the PVN^GLP-1R^→DVC pathway directly regulates blood glucose metabolism. We conducted glucose tolerance tests (GTT) in the same group of animals with DREADDs activation by CNO but found no significant effects on GTT (**Extended Data Fig. 3d**). These results indicate that the activity of a subpopulation of PVN^GLP-1R^→VC neurons in sufficient to selectively regulate food intake behavior.

## PVN^GLP-1R^→DVC neuronal activity response to food is dependent on energy state

PVN^GLP-1R^ neurons are heterogenous but show increased activity during food access^20^. To test whether PVN^GLP-1R^→DVC subpopulation of PVN neurons respond to food, we utilized fiber photometry to record their real-time activity dynamics^20–22^. To allow robust expression of the genetically encoded Ca^2+^ sensor GCaMP in PVN^GLP-1R^→DVC neurons, we injected AAVrg-EF1a-DIO-Flpo in the DVC and AAV-hSyn-fDIO-GCaMP6f in the PVN of GLP-1R-ires-Cre mice (Fig. 2f&g). Using fiber photometry in behaving animals, we found that these neurons showed significant increases in activity during grooming and in response to tail pick stress (**Extended Data Fig. 4a-d**). Interestingly, we also observed that the relative basal activity of these neurons was reduced when animals underwent overnight fasting (i.e. hunger or energy deficiency state) compared with *ad libitum* feeding (i.e. energy sufficient state), suggesting that PVN^GLP-1R^→DVC neuronal activity is correlated with energy state (energy sufficient *vs.* deficient) (Fig. 2h, i). We next asked whether PVN^GLP-1R^→DVC neuronal activity is affected by food presentation in an energy state-dependent manner. We presented chow pellets or non-edible objects to mice in two different metabolic states: energy deficient (fasted) or energy replete (*ad libitum* fed). Interestingly, food presentation to fasted animals significantly increased PVN^GLP-1R^→DVC neuronal activity, but not in the energy replete (i.e. fed) state. In contrast, the introduction of a non-food object had no effect on PVN^GLP-1R^→DVC activity (Fig. 2j&k). Furthermore, we also investigated whether food accessibility is necessary for the induction of PVN^GLP-1R^→DVC activity. Mice were presented with a chow pellet confined within an enclosed tea ball, allowing them to see and smell the food directly without consuming it. Interestingly, PVN^GLP-1R^→DVC neuronal activity in fasted mice, but not fed mice, showed a prominent increase in neuronal activity. Meanwhile, non-edible objects did not induce neuronal activity changes (**Extended Data Fig. 4e&f**). Overall, these data strongly suggest that PVN^GLP-1R^→DVC neuronal responses to food stimulation are energy state-dependent.

## State-dependent synaptic plasticity in the PVN^GLP-1R^→DVC neuronal circuit

Intake of large volumes of highly palatable diet activates GLP-1 releasing neurons^23^, increases immediate early gene cFos expression in the PVN, and PVN^GLP-1R^ neurons’ spontaneous action potential firing rate^20^. Given that food intake-induced neuronal activity in the PVN^GLP-1R^→DVC pathway is dependent on energy state (Fig. 2h–j), we hypothesized that GLP-1 regulation of synaptic transmission within this neural circuit (Fig. 1g–n) is also energy state-dependent. To test this hypothesis, we again conducted patch clamp recordings to interrogate the changes in electrophysiological activity between fasted (energy deficient) and well-fed (energy replete) states.

We first probed the intrinsic membrane properties of the PVN^GLP-1R^ neurons that project to the DVC after fasting. We injected AAVrg-hSyn-DIO-EGFP in the DVC of GLP-1R-ires-Cre mice and conducted patch clamp recordings from DVC-projecting PVN^GLP-1R^ neurons (**Extended Data Fig. 5a**). Despite overnight fasting, no detectable differences in membrane capacitance, membrane resistance, or resting membrane potential (RMP) were found (**Extended Data Fig. 5b-d**). We also measured the spontaneous activity and input-output relationship (current injection and action potential numbers) of PVN^GLP-1R^→DVC neurons but found no differences between fasted and well-fed conditions (**Extended Data Fig. 5e-j**). These data indicate that the membrane excitability of PVN^GLP-1R^ neurons projecting to DVC was unaffected by energy states.

**Figure 3:**
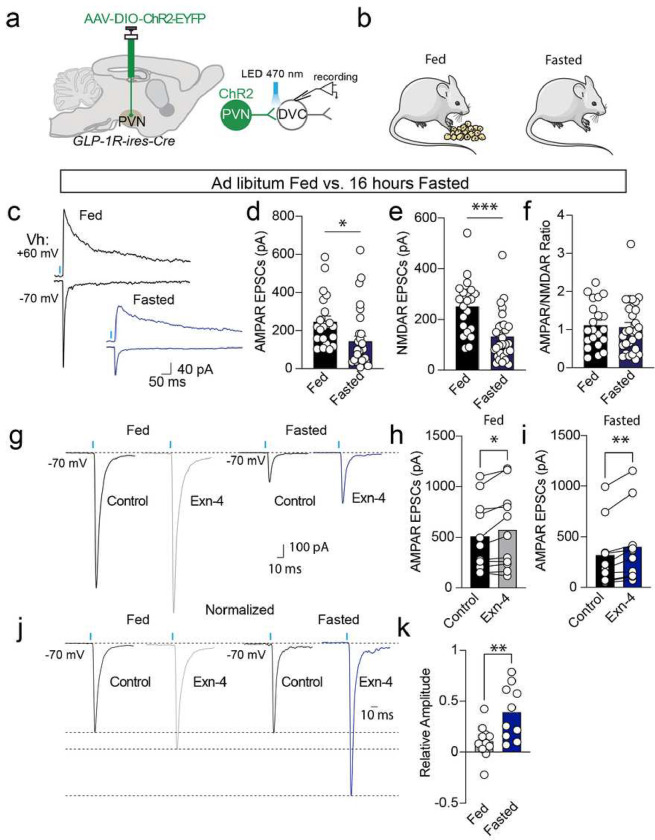
State-dependent synaptic plasticity of PVNGLP-1R→DVC neurons. **a-b**. Experimental paradigm for AAV-DIO-Chr2-EYFP injection and electrophysiology experiments. **c-f**. Representative traces and quantification of AMPAR-mediated oEPSCs (Fed n=21 cells/3 mice, Fasted n=30 cells/4 mice), NMDAR EPSCs (Fed n=21 cells/3 mice, Fasted n=29 cells/4 mice), and AMPAR/NMDAR EPSC ratio (Fed n=21 cells /3 mice, Fasted n=29 cells/4 mice) under different conditions. **g-i**. Representative traces and quantification of AMPAR oEPSCs witho or without Exn-4 under different energy states (Fed n=11 cells/4 mice, Fasted n=10 cells/3 mice). **j-k**. Representative traces and quantification of normalized AMPAR-mediated oEPSCs with or without Exn-4 under different energy states (Fed n=11 cells/4 mice, Fasted n=10 cells/3 mice). Data are presented as mean ± SEM and n numbers are indicated in each plot. Student’s t-tests are applied to (**d-f**) and (**k**), and paired student’s t-tests are applied to (**h**) and (**i**). *p< 0.05; **p<0.01, ***p<0.001.

Next, we investigated synaptic release from PVN^GLP-1R^→DVC neurons after overnight fasting (Fig. 3a&b). We found a remarkable decrease in oEPSCs mediated by both AMPAR (recorded at a holding potential of −70 mV) and NMDAR (recorded at a holding potential of +60 mV, measured at 50ms after optical stimulation) with no change in AMPAR/NMDAR oEPSC ratio in fasted animals (Fig. 3d–f). These data suggested that the presynaptic release probability of glutamate at the PVN^GLP-1R^→DVC synapse is reduced when body energy is low. Because circulating GLP-1 levels are circadian and postprandially related^24–27^, it is conceivable that after overnight fasting, circulating GLP-1 levels are lower than under *ad libitum* feeding (energy replete state). Since GLP-1R signaling augments synaptic strength at this synapse (Fig. 1), we therefore hypothesized that synaptic transmission at this synapse will be more sensitive under the hunger state because of low GLP-1 levels. Consistent with this hypothesis, we found a larger relative increase in synaptic transmission under the hunger state (Fig. 3g–k). This result highlights the regulatory effects of GLP-1 on synaptic transmission and the energy state-dependent dynamic synaptic strength changes contributing to energy replenishment to maintain energy homeostasis.

## High-fat diet (HFD)-induced obesity (DIO) blunted the neuronal activity of PVN^GLP-1R^→DVC neurons

Energy homeostasis is perturbed in obesity, and we hypothesize that energy state-dependent synaptic plasticity at the PVN^GLP-1R^→DVC synapse would be disrupted in the obese state. To test this hypothesis, we investigated synaptic transmission in a DIO mouse model.

To label the PVN^GLP-1R^→DVC neurons, we injected AAVrg-hSyn-DIO-EGFP in the DVC of GLP-1R-ires-Cre mice. After 12 weeks of HFD/control diet exposure (**Extended Data Fig. 6a&b**), we first examined the intrinsic properties of PVN^GLP-1R^→DVC neurons. We found minor changes in the membrane properties including RMP but no change in cell capacitance (**Extended Data Fig. 6c&d**). We also observed a slightly increased trend of spontaneous action potential firing frequency but no changes in the input-output relationship of the current injection and induced action potential firing (**Extended Data Fig. 6e-j**). Thus, excitability is only modestly affected in these cells in DIO animals.

**Figure 4:**
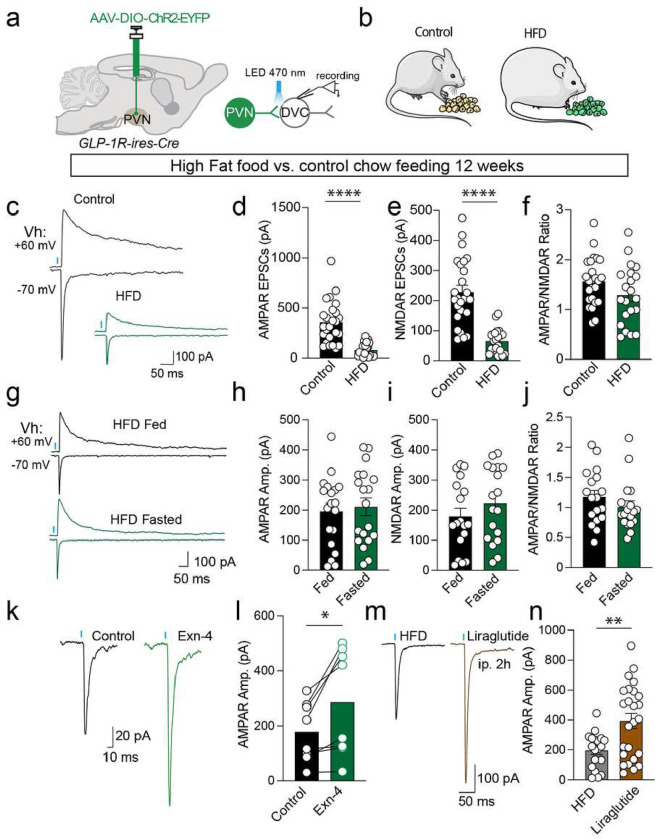
High-fat diet (HFD) induced obesity blunts the state-dependent synaptic plasticity of PVNGLP-1R→DVC neurons. **a-b**. Experimental paradigm for AAV-DIO-Chr2-EYFP injection and electrophysiology experiments. **c-f**. Representative traces and quantification of AMPAR-mediated oEPSCs (Control n=25 cells/3 mice, HFD n=23 cells/3 mice), NMDAR-oEPSCs (Control n=25 cells/3 mice, HFD n=21 cells/3 mice), and AMPAR/NMDAR EPSC ratio (Control n=25 cells/3 mice, HFD n=21 cells/3 mice) in control or HFD animal. **g-i**. Representative traces and quantification of AMPAR oEPSCs (Fed n=19 cells/3 mice, Fasted n=19 cells/3 mice), NMDAR oEPSCs (Fed n=18 cells/3 mice, Fasted n=18 cells/3 mice), and AMPAR/NMDAR EPSCs ratio (Fed n=18 cells/3 mice, Fasted n=18 cells/3 mice) under different energy states in HFD-induced obese animals. **k-l**. Representative traces and quantification of AMPAR-mediated oEPSCs before and after application of Exn-4 in HFD-induced obese animals (n=8 cells/7 mice). **m-n**. Representative traces and quantification of AMPAR-mediated oEPSCs after control or Liraglutide i.p. 2h injection (control n=19 cells/3 mice, Liraglutide n=25 cells/3 mice) in HFD obese mice. Data are presented as mean ± SEM and sample sizes are indicated in each plot; Student’s t-tests are applied to (**d-j**) and (**n**), and paired student’s t-tests are applied to (**l**). *p< 0.05; **p<0.01, ***p<0.001, ***p<0.0001.

We then investigated whether the excitatory synaptic strengths from PVN^GLP-1R^→DVC neurons are altered under DIO, a chronic overnutrition state (Fig. 4a&b). We found a significant decrease in both AMPAR and NMDAR-mediated oEPSCs with no change in AMPAR/NMDAR EPSC ratio in the HFD-fed animals compared with those fed the control diet (Fig. 4c–f). These data indicate that the presynaptic release of glutamate at the PVN^GLP-1R^→DVC synapse is reduced in DIO subjects compared to controls. Since fasting overnight had a significant impact on PVN^GLP-1R^→DVC synaptic release in normal body weight animals (Fig. 3a–f), we tested if we could observe a similar impact of energy status on synaptic strength at this synapse in DIO subjects. However, we found no change in oEPSCs mediated by AMPAR and NMDAR under energy deficient, i.e. fasted, and energy repleted, i.e. fed, states (Fig. 4g–j). These data suggest that the dynamic changes in synaptic strength (presynaptic release probabilities) at the PVN^GLP-1R^→DVC synapse are disrupted in DIO mice.

Since GLP-1R agonists are effective in obese humans in reducing appetite and body weight^4^, we hypothesized that GLP-1R signaling is still intact in DIO animals to regulate synaptic transmission. Indeed, the application of Exn-4 significantly increased PVN^GLP-1R^→DVC synaptic release in ex vivo slice recordings (Fig. 4k&l). We further hypothesized that systemically administered GLP-1R agonists, such as liraglutide^28^, may exert actions on the PVN^GLP-1R^→DVC pathway. To this end, we injected 400 μg/kg liraglutide intraperitoneally in DIO animals and conducted brain slice recordings after 2 h post-injection. We found liraglutide-injected DIO animals show enhanced PVN^GLP-1R^→DVC oEPSCs mediated by AMPAR and NMDAR (Fig. 4m&n and **Extended Data Fig. 7**). These results indicate that GLP-1R mediated signaling is intact in DIO animals and systemic administration of GLP-1 analogs may suppress feeding by augmenting PVN^GLP-1R^→DVC synaptic strength.

Overall, these data suggest that within the PVN^GLP-1R^→DVC circuit, the synaptic strengths are dynamically changing with energy state (i.e. stronger synaptic transmission when energy replete, weaker when energy depleted (Fig. 3)). This dynamic synaptic plasticity is disrupted in DIO mice, an overly nutritional state (Fig. 4g–j), but nevertheless can be rescued by GLP-1R agonists (Fig. 4k–n).

## Ablation of presynaptic GLP-1R and inactivation of PVN^GLP-1R^→DVC neuronal synaptic release cause obesity

**Figure 5:**
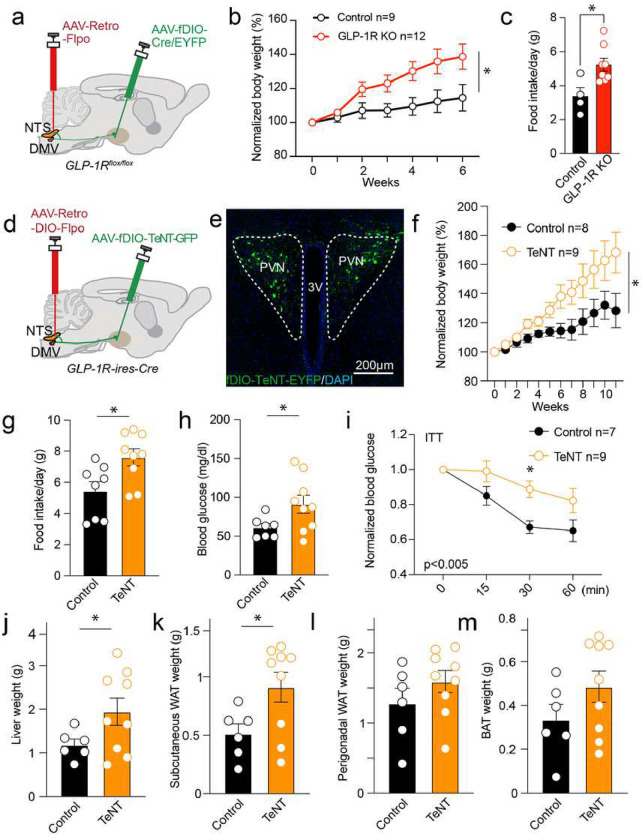
Chronic perturbation of PVNGLP-1R→DVC neurons induces obesity. **a**. Experimental paradigm for the depletion of PVN^GLP-1R^**→**DVC neurons GLP-1R using AAV-Cre virus injection. **b**. Quantification of body weight gain after depletion of PVN^GLP-1R^**→**DVC neurons GLP-1R (Control n=9 mice, Knockout n=12 mice). **c**. Quantification of daily food intake after GLP-1R ablation in PVN^GLP-1R^**→**DVC neurons compared to control animals (Control n=4 mice, Knockout n=8 mice). **d**. Experimental paradigm for the chronic inactivation of PVN^GLP-1R^**→**DVC neurons synaptic release. **e**. Representative image of the PVN showing the expression of TeNT-GFP in the PVN^GLP-1R^**→**DVC neurons. **f**. Quantification of body weight gain after expressing TeNT/Control in PVN^GLP-1R^**→**DVC neurons (Control n= 7–8 mice, TeNT n=9 mice). **g**. Quantification of daily food intake after expressing TeNT in PVN^GLP-1R^**→**DVC neurons as compared to control animals (control n=8 mice, TeNT n=9 mice). **h**. Fasting glucose levels (Control n=7 mice, TeNT n=9 mice). **i**. Insulin tolerance test-induced glucose level changes (Control n=7 mice, TeNT n=9 mice). **j-m** Liver, subcutaneous WAT, perigonadal WAT, and BAT weight in TeNT-injected and control animals (Control n=6 mice, TeNT n=9 mice). Data are presented as mean ± SEM and sample sizes are indicated in each plot. Two-way ANOVA, with Geisser-Greenhouse correction, is applied to (**b**), (**f**), and (**i**); Student’s t-tests are applied to (**c**), (**g-h**), and (**j-m**). *p< 0.05; **p<0.01, ***p<0.001, ***p<0.0001.

Having demonstrated that GLP-1R signaling regulates synaptic strength in the PVN^GLP-1R^→DVC descending circuit and the involvement of this in controlling feeding, we asked whether GLP-1Rs in this circuit are necessary for maintaining energy homeostasis. To achieve specific ablation of PVN^GLP-1R^→DVC neurons, we injected AAVrg-FLPo-WPRE-hGHpA in the DVC and AAV-EF1a-fDIO-Cre in the PVN in GLP-1R^flox/flox^ mice^29^. This strategy allows Cre recombinase expression and thus knockout (KO) of GLP-1R specifically in DVC-projecting PVN GLP-1R neurons (Fig. 5a). Compared to control animals expressing GFP, PVN^GLP-1R^→DVC KO animals exhibited significant body weight gain and increased daily normal chow intake (Fig. 5b&c). These results suggest that GLP-1R signaling in this pathway is vital to controlling energy homeostasis, likely via regulating synaptic release at this synapse.

To directly test this possibility, we expressed tetanus toxin (TeNT) light chain in PVN^GLP-1R^ neurons that project to the DVC to irreversibly silence synaptic release by cleaving the vesicle protein synaptobrevin^30^. In GLP-1R-ires-Cre mice, we injected AAVrg-DIO-FLPo in the DVC and AAV-CMV-fDIO-TeNT or control virus in the PVN (Fig. 5d&e). The TeNT-expressing mice gained significantly more body weight and exhibited increased daily food intake compared to controls (Fig. 5f&g). We further examined glucose metabolism at 13 weeks after inactivating PVN^GLP-1R^→DVC synaptic transmission and found that the inactivation group exhibited elevated fasting blood glucose levels, and decreased insulin tolerance (ITT) (Fig. 5h&i). Since chemogenetic activation of PVN^GLP-1R^→DVC neurons did not affect GTT (**Extended Data Fig. 3d)**, the blood glucose impairment was likely a consequence of obesity induced by inactivation. Moreover, an altered respiratory exchange ratio (RER) was also observed (**Extended Data Fig. 8**). The TeNT group also displayed an increase in liver and subcutaneous white adipose tissue (WAT) weight, while the perigonadal WAT and brown adipose tissue (BAT) weight remained unchanged (Fig. 5j–m).

Collectively, these data strongly indicate that GLP-1R signaling and synaptic transmission in the hypothalamic-brain stem descending circuit, i.e. the PVN^GLP-1R^→DVC, contribute significantly to energy balance regulation.

## Discussion

GLP-1 analogs are effective in treating type 2 diabetes and obesity, but the physiological functions and the pharmacological targets in the brain that mediate these important effects are not fully understood^4^. Therefore, unraveling the CNS function of GLP-1R signaling is crucial. This study reveals a hypothalamus-brain stem descending pathway as an important mediator in GLP-1R signaling, feeding behavior, and body weight homeostasis. We find that GLP-1R signaling in the PVN^GLP-1R^→DVC descending pathway regulates synaptic plasticity to maintain energy homeostasis. Disruption of synaptic transmission or ablation of GLP-1R in this circuit causes obesity. In DIO conditions, the state-dependent synaptic plasticity in the circuit is disrupted but still responsive to GLP-1. Our study highlights that this dynamic synaptic plasticity in the PVN^GLP-1R^→DVC descending circuit regulated by GLP-1 is crucial for maintaining energy homeostasis.

While previous studies have demonstrated a role of oxytocin in the PVN to brain stem pathway^31^, we show a role for presynaptic GLP-1R signaling in this pathway for the first time in this study. Administering GLP-1R antagonists or genetic manipulation of GLP-1R expression supports a role for both hypothalamic and hindbrain GLP-1R in the physiological control of food intake^28,32^. However, central and peripheral GLP-1 systems suppress eating via independent gut-brain circuits^7^. Clinical studies suggest that GLP-1 reduces postprandial glycemic excursions but no longer inhibits food intake or the rate of gastric emptying in non-diabetic normal-weight men after truncal vagotomy and pyloroplasty^33^. Therefore, hindbrain vagal circuits must be critical for endogenous GLP-1 action and the pharmacological responses to GLP-1R agonists. Nevertheless, it is unclear how GLP-1R signaling translates to feeding regulation via the hypothalamus-brain stem descending pathway. In this study, we found that GLP-1R-mediated signaling in the PVN^GLP-1R^→DVC circuit is required for maintaining energy homeostasis. We showed that 1) GLP-1 regulates PVN^GLP-1R^→DVC glutamate release via the PKA pathway in the presynaptic compartment (Fig. 1); 2) ablation of GLP-1R in the presynaptic neurons in the PVN^GLP-1R^→DVC circuit causes obesity (Fig. 5a–c); 3) blockade of synaptic transmission in the PVN^GLP-1R^→DVC circuit causes obesity and glucose metabolism deficits (Fig. 5d–m). These data demonstrate that the descending PVN^GLP-1R^→DVC circuit is a crucial target (both necessary and sufficient) for endogenous GLP-1 and/or pharmacological GLP-1 agonists. We recognize the complexity of neuronal subtypes in the DVC^14^: the cholinergic neurons in the DMV^34^, the tyrosine-hydroxylase positive cells in the AP^35^, NTS^36^, and the other cell types including the GLP-1 producing and GLP-1R positive neurons in the NTS^10,23^. Whether or not PVN^GLP-1R^ neurons project to NTS GLP-1-producing neurons to form a feedback loop remains to be elucidated in future studies.

Circulating GLP-1 levels, mainly from the gut L-type cells, increase postprandially^25,26,37^. In the brain, GLP-1 released from NTS neurons is likely the main source of GLP-1, and food intake activates these neurons; therefore, brain GLP-1 likely will also increase postprandially^38,39^. NTS GLP-1 neurons were shown to be activated by mechanical feedback from the gut, tracked food intake, and promoted long-lasting satiety^3^. We showed in this study that synaptic transmission in the PVN^GLP-1R^→DVC circuit is crucial for energy metabolism and found that synaptic plasticity in this circuit is dynamically regulated by energy states (Fig. 3): when energy is replete, PVN^GLP-1R^→DVC synaptic release is strong, therefore suppressing further food intake. When energy is deficient (i.e. in hunger), PVN^GLP-1R^→DVC synaptic release is weak, therefore allowing food intake. We further demonstrated that synaptic plasticity in this circuit is regulated by GLP-1, which has a more profound impact on synaptic release under energy-deficient states (e.g., after overnight fasting). In *ad libitum*-fed animals, there were higher GLP-1 levels compared to the fasted state, which impacted the synapse and precluded further increases in synaptic strength when an exogenous GLP-1 analog was applied. We speculate that the dynamic changes of brain GLP-1 with mealtime are the main regulator of synaptic plasticity in the PVN^GLP-1R^→DVC circuit, and changes in PVN^GLP-1R^→DVC synaptic strength regulate food intake behavior.

It has been reported that HFD-induced obesity remodels neurocircuits in the hypothalamic arcuate nucleus^40^ and the lateral hypothalamus^41^. In our study, we observed a marked reduction in PVN^GLP-1R^→DVC synaptic strength in DIO animals (Fig. 4c–f). This is somewhat surprising—since it has been reported that fasting GLP-1 levels are increased in obesity^42^, we would expect that if GLP-1 levels are higher in the brains of DIO animals, synaptic transmission would be stronger. Another alternative explanation is that the GLP-1R is desensitized due to the higher GLP-1 levels in the obese subjects. However, this appears not to be the case since both in vitro applied GLP-1 analog Exn-4 and systematically applied liraglutide facilitated synaptic release in the PVN^GLP-1R^→DVC pathway (Fig. 4k–n), suggesting GLP-1R mediated signaling appeared to be intact in obese mice. Moreover, there was no significant change in these synaptic responses between ad libitum fed and overnight fasting in DIO animals (Fig. 4g–i). Under normal body weight, the synaptic strengths are likely regulated by different brain GLP-1 levels at different energy states, i.e. stronger synaptic releases at the PVN^GLP-1R^→DVC synapse with ad libitum fed, but weaker synaptic strength after fasting (energy deficient, lower GLP-1). Therefore, we speculate endogenous GLP-1 production or release in obese subjects is defective, consistent with a previous report demonstrating oral glucose-induced GLP-1 release reduction in obese subjects^43^. This also explains why GLP-1 analog therapeutics are effective in obese patients. However, the defective production of GLP-1 in obese subjects would disrupt the dynamic changes of GLP-1 levels with energy states and thus fail to regulate synaptic transmission in the PVN^GLP-1R^→DVC pathway according to the energy states (deficient vs. repleted). Failure to appropriately regulate synaptic plasticity according to energy state is proposed to underlie overeating. We thus argue that the dynamic changes in GLP-1 levels in the brain to regulate synaptic strength depending on energy states and energy needs are crucial in feeding regulation. We thus propose that therapeutics mimicking the endogenous dynamic changes of GLP-1 levels may be more effective and better for patient health.

## Methods

### Animals.

All studies and procedures involving mice were approved by the Rutgers University Institutional Animal Care and Use Committee (IACUC) and by the National Institute of Health (NIH) guidelines. The animals used in this study were 5–20 weeks old, housed, and bred in the Child Health Institute of New Jersey animal facility. Homozygous GLP-1R-ires-Cre^44^, and GLP-1R-flox^28^ mice were used in the present study. Mice were housed in a 12-hour light–dark cycle (6:00–18:00) with *ad libitum* access to food and water unless otherwise indicated (fasting studies and HFD studies). In all cases, when possible, mice were randomized according to body weight in each experimental group. The investigators were blinded to the treatment.

### AAV virus and stereotaxic injections.

The AAV-viruses used in this study include: AAV2-Y444F-CAG-DIO-mWGA-mCherry (VIROVEK, Cat# 117-UCSF86), pAAV-EF1a-double-floxed-hChR2 (H134R)-EYFP-WPRE-HGHpA (Addgene, Cat# 20298-AAV9), pAAV-hSyn-DIO-EGFP (Addgene, Cat# 50457-AAVrg), AAV pEF1a-DIO-FLPo-WPRE-hGHpA (Addgene, Cat# 87306-AAVrg), pAAV-EF1a-Flpo (Addgene, Cat# 55637-AAVrg), pAAV-EF1a-fDIO-Cre (Addgene, Cat# 121675-AAV9), pAAV-Ef1a-fDIO-GCaMP6f (Addgene, Cat# 128315-AAV1), pAAV-hSyn-fDIO-hM3D(Gq)-mCherry-WPREpA (Addgene, Cat# 154868-AAV8), pAAV-Ef1a-fDIO mCherry (Addgene, Cat# 55641-AAV1), and pAAV-CMV-fDIO-TeNT-EYFP (obtained from Dr. Wei Xu at UT Southwestern Medical Center). AAV virus was injected using a Nanoject III^®^ with 1nl/s speed stereotactically in 5–7 weeks old male mice under isoflurane anesthesia. The injection coordinates used for the DVC were: AP: −7.5 mm from bregma; mediolateral (ML): ± 0.5 mm; dorsoventral (DV): −4.9 mm. The injection coordinates used for the PVN were: AP: −0.92 mm from bregma; ML: ± 0.18 mm; DV: −4.75 mm. Viral-mediated gene expression was allowed for 3–4 weeks before experimental manipulation. Injection sites were confirmed post hoc by cutting brain sections and inspecting them under a stereoscope or microscope in all animals reported in this study.

### Histology and Immunohistochemistry assay.

Mice were anesthetized with Euthasol and transcardially perfused with 4% PFA in PBS, pH 7.4. The brains were postfixed in 4% PFA overnight and then moved to 30% sucrose for at least 24 hours. Coronal brain cryosections (50 μm) were cut thereafter. For immunohistochemistry experiments, sections were incubated in blocking buffer (4% BSA, 1% goat serum, 0.2% Triton X-100 in PBS) for 1 hour and then incubated overnight with the primary antibody at 4°C. The next day, brain sections were washed with blocking buffer at least 3 times, incubated with secondary antibody at room temperature for 2 hours, and washed with PBS. Brain sections were then mounted onto glass slides with Fluoroshield (with or without DAPI). Images were acquired with a Zeiss LSM700 confocal microscope. Z-stack images captured the entire thickness of the section at 10 μm steps for images taken with a 20 × objective (N.A. 0.8). PVN GLP-1R-expressing neurons and their axon terminals were labeled with AAV2-Y444F-CAG-DIO-mWGA-mCherry or pAAV-EF1a-double-floxed-hChR2 (H134R)-EYFP-WPRE-HGHpA by injection in the PVN of GLP-1R-ires-Cre animals. In retrograde tracing experiments, PVN^GLP-1R^→DVC neurons were labeled by injecting pAAVrg-hSyn-DIO-EGFP in the DVC of GLP-1R-ires-Cre animals.

### Antibodies.

The primary antibodies used in this study were Rabbit anti-CHAT (1:500, Sigma, Cat# 226 008), Chicken anti-GFP (1:500, Enquire, Cat# CGFP-45A) and Rabbit anti-RFP (1:500, Rockland Antibodies and Assays, Cat# 600–401-379).

### Electrophysiology.

Brain slice electrophysiology was conducted as described elsewhere^18^. Mice were anesthetized and decapitated, and brains were removed and quickly immersed in the cold (4°C) oxygenated cutting solution containing (in mM): 50 sucrose, 2.5 KCl, 0.625 CaCl_2_, 1.2 MgCl_2_, 1.25 NaH2PO_4_, 25 NaHCO_3_, and 2.5 glucose. Coronal cerebral cortex slices, 300 mm in thickness, were cut using a vibratome (VT 1200S; Leica). Brain slices were collected in artificial cerebrospinal fluid (ACSF) and bubbled with 5%CO_2_ and 95%O_2_. The ACSF contained (in mM): 125 NaCl, 2.5 KCl, 2.5 CaCl_2_, 1.2 MgCl_2_, 1.25 NaH2PO_4_, 26 NaHCO_3_, and 2.5 glucose. After 1 hour of recovery, slices were transferred to a recording chamber and constantly perfused with bath solution (33°C) at a flow rate of 2 ml/min. To record EPSCs, picrotoxin (50 mM, Sigma) was added to block IPSCs mediated by GABAA receptors. GLP-1 analog Exn-4 (100 nM, Tocris) was added to the bath solution to activate GLP-1Rs on PVN^GLP−1^→DVC neurons. Patch pipettes with a resistance of 5–8 MΩ were made from borosilicate glass (World Precision Instruments) with a pipette puller (PC-10, Narishige) and filled with the pipette solution containing (in mM): 126 K-Gluconate, 4 KCl, 10 HEPES, 4 Mg-ATP, 0.3 Na2-GTP, 10 phosphocreatine (pH to 7.2 with KOH) for current and voltage-clamp recordings. After the whole-cell patch clamp was achieved, spontaneous EPSCs were recorded under a voltage clamp at −70 mV. All data were analyzed offline using ClampFit 10.2 (Molecular Devices, USA) software.

### Fiber Photometry.

We injected AAVrg-EF1a-DIO-Flpo in the DVC and AAV-hSyn-fDIO-GCaMP6f in the PVN of GLP-1R-ires-Cre mice. GcaMP6s signal was collected through an optical fiber unilaterally implanted above the PVN (diameter: 200 μm; length: 5.0 mm; NA: 0.37). Recordings took place at least 3 weeks after virus delivery. A continuous blue LED at 465 nm and UV at 405 nm served as an excitation light source (Thorlabs), modulated at 211hz and 333hz respectively, and delivered to a filtered minicube (FMC6, Doric Lenses) before connecting through optic fibers to a rotary joint to allow for movement. GcaMP calcium GFP signals were detected by a visible femtowatt photoreceiver (Tucker- Davis Technologies, Model 2151) with the gain set to DC low. The light was then converted to electrical signals and demodulated by a real-time processor (Tucker- Davis Technologies, RZ5P).

Data was collected through the software Synapse (TDT) and exported via Browser (TDT) Fluorescence emission was captured by Tucker-Davis Technologies hardware at about 1 kHz. Collected data were then processed in Python. Fluorescence data was downsampled to 10 Hz and smoothed with a 100 ms rolling average filter. The isosbestic 405 nm data channel was linearly scaled to the 465 nm channel and used as f0 to calculate ΔF/F = (f − f0)/f0. The change in fluorescence was normalized to the mean change of the whole recording. This data stream was then aligned around each behavioral event, with the baseline mean of 10–30 seconds before the event was subtracted out. After this correction, the average and standard error across all events was obtained and visualized. The area under the curve was calculated using event-aligned and background-subtracted ΔF/F (tail pick: 10s pre vs. post; grooming: 60s pre vs. post; food-related events: AUC of 3 minutes post subtracted by AUC of 1-minute pre-event). Peak frequency and amplitude were calculated using Scipy’s find_peaks function (1s distance, prominence of 1.4). Data, as presented in figures (not for statistics), were then smoothed and downsampled further.

Sensory detection experiments were conducted as described in ^20^. Experiments were conducted in the afternoon. Mice were fasted for 12 to 16 hours by food deprivation. They were habituated to the open arena for at least 30 minutes before beginning of experimentation. For presentation experiments, photometry data was obtained as mice was presented with either a chow pellet or non-edible object (falcon tube). For tea ball presentation experiments, mice were first presented with either chow or an object in an inaccessible tea ball for 20 minutes. Then, the tea ball was opened, and the contents were accessible for 20 minutes. For all these experiments, 10 minutes of baseline photometry data was first obtained.

### Chemogenetics.

For the activation of PVN-to-DVC GLP-1R neuron, we injected 250 nL of AAV-pEF1a-DIO-FLPo-WPRE-hGHpA (Cat# 87306-AAVrg) in the DVC (AP: −7.5 mm from bregma; lateral (L): ±0.5 mm, dorsal-ventral (DV): −4.9–5.0 mm.) and pAAV-hSyn-fDIO-hM3D(Gq)-mCherry-WPREpA (Cat# 154868-AAV8) or pAAV-Ef1a-fDIO mCherry (Cat# 114471-AAV8) in PVN (AP: −0.92 mm from bregma; lateral (L): ±0.18 mm, dorsal-ventral (DV): −4.7–4.8 mm).

### Glucose Tolerance Test (GTT).

GTT was conducted in overnight fasted mice as described elsewhere ^45^. At Time 0, blood glucose was measured to set a baseline. 1 mg/kg body weight CNO was injected intra-peritoneally. After 30 minutes, 1 g/kg body weight of 20% dextrose solution was injected i.p., and blood samples were collected from the tail vein at 15, 30, 60, and 120 minutes. For GTT, injection volume was calculated as mentioned below:

$$Injection\;volume\;(\mu L)=\;mouse\;weight\;(Kg)\times \frac{\frac{2g}{kg}glucose\;}{0.2\;g/ml}=mouse\;weight\;(kg)\times 10ml/kg\;glucose=\;mouse\;weight\;(g)\times 10$$

### Insulin Tolerance Test (ITT).

ITT was conducted in mice after fasting of 6 hours as described elsewhere ^45^. At Time 0, blood glucose was measured to set a baseline. insulin (1 unit/kg ITT) was injected i.p., blood samples were collected from the tail vein at 15, 30, and 60 minutes. The plasma glucose was measured by a glucometer (FreeStyle Lite system, Abbott Diabetes Care CA, USA).

$$Injection\;volume\;(\mu L)=\;mouse\;weight\;\;(kg)\times \frac{\frac{1unit\;}{kg}insulin\;}{0.1unit/ml}=mouse\;weight\;(kg)\times 10ml/kg\;insulin=\;mouse\;weight\;(g)\times 10$$

### Comprehensive Lab Animal Monitoring System (CLAMS) assay.

Mice were placed into metabolic phenotyping cages (Columbus Instruments, OH, USA). Data was recorded every 15 minutes. Data was collected for 3 days (both light and dark cycles) after 2 days of habituation to single housing within the cages.

### Food Intake.

Mice were singly housed before the experiment on a 12 h light/dark cycle with *ad libitum* access to water and were fasted overnight for 12 hours (9 pm-9 am), 0.5 g of food was given to reduce animal anxiety. The following day, each animal’s weight was measured, CNO was prepared, and 1mg/kg BW CNO was injected *i.p.* into each mouse. After 30 minutes, food was added to their cage. Standard chow intake was measured at t=0,0.5,1,2,3 and 24 hours. After the experiment, animals were grouped into their respective cages.

### Open Field Locomotor Activity.

The open field test was performed as previously described ^46^. Briefly, mice were placed in the middle of a custom-made 45 × 45 cm square open field arena for 10 minutes. The time spent in the center and total distance traveled were quantified using DeepLabCut version 2.2 and region of interest selection ^47^. The pose estimation model (ResNet-50-based with default parameters) was trained for 600,000 iterations on 200 frames of open field arena data extracted from 10 videos. 95% of these frames were used for training and 5% for testing. Estimation data was processed using custom software adapted from DLC2Kinematics and region of interest tools. The inner three-fifths of the arena was defined as the center zone. Time in the center zone was calculated using the center of the mouse’s body. During analysis, investigators were also blinded to the experimental group.

### Light-dark box.

The light/dark box is viewed as an ethological model of anxiety, placing into competition the drives to remain safe and to explore novel environments. Mice were placed in the light side of a commercial light-dark box apparatus (AlfaSci) consisting of one dark and one illuminated compartment connected by a door. Time spent in each zone, latency to enter the dark zone, and number of entries were measured for 10 minutes.

Ablation of GLP-1R in PVN^GLP-1R^→DVC neurons.

To achieve specific ablation of PVN^GLP-1R^→DVC neurons, we injected AAVrg-FLPo-WPRE-hGHpA in the DVC and AAV-EF1a-fDIO-Cre in the PVN in GLP-1R^flox/flox^ mice. Body weight was monitored every week post-surgery (week 0). On week 6, animals were separated into individual cages to adapt to their surroundings for one day. Food intake was measured from day 3 to day 8.

### Tetanus toxin (TeNT) light chain inactivation of synaptic transmission.

In GLP-1R-ires-Cre mice, we injected AAVrg-DIO-FLPo in the DVC and pAAV-CMV-fDIO-TeNT-EYFP or control virus in the PVN. Body weight was monitored every week post-surgery (week 0). On week 7, animals were separated into individual cages to adapt to their surroundings for one day. Food intake was measured from day 3 to day 8. During week 12, all the animals were placed into metabolic cages (Columbus Instruments, Comprehensive Lab Animal Monitoring System (CLAMS), OH, USA) to adapt to their surroundings 12 hr before measurement. All metabolic data were collected in both day and night phases.

### Statistical information.

Statistical analysis was performed using Excel (version 2019). In addition, statistical analysis for blood glucose and food intake behavior was also analyzed using GraphPad Prism 9.0. All the data is presented as mean ± standard error of the mean (SEM). Most of the data were analyzed using two-sided Student’s t-test, and One-way ANOVA. Repeated-measurement 2-way ANOVA and Geisser-Greenhouse’s epsilon correction were used for all time-dependent experiments. Non-significant results (p>0.05) are not displayed. A p-value of less than 0.05 was considered statistically significant. All the details of the experiments can be found in the figure legends. All data values are presented as mean ± SEM.

## Data Availability

The data and datasets used and/or analyzed during the current study are available from the corresponding authors upon reasonable request.
